# Starved epithelial cells uptake extracellular matrix for survival

**DOI:** 10.1038/ncomms13989

**Published:** 2017-01-10

**Authors:** Taru Muranen, Marcin P. Iwanicki, Natasha L. Curry, Julie Hwang, Cory D. DuBois, Jonathan L. Coloff, Daniel S. Hitchcock, Clary B. Clish, Joan S. Brugge, Nada Y. Kalaany

**Affiliations:** 1 Department of Cell Biology, Harvard Medical School, Boston, Massachusetts 02115, USA; 2 Division of Endocrinology, Center for Basic and Translational Obesity Research, Boston Children's Hospital, Boston, Massachusetts 02115, USA; 3 Broad Institute of Harvard and MIT, Cambridge, Massachusetts 02142, USA; 4 Department of Pediatrics, Harvard Medical School, Boston, Massachusetts 02115, USA

## Abstract

Extracellular matrix adhesion is required for normal epithelial cell survival, nutrient uptake and metabolism. This requirement can be overcome by oncogene activation. Interestingly, inhibition of PI3K/mTOR leads to apoptosis of matrix-detached, but not matrix-attached cancer cells, suggesting that matrix-attached cells use alternate mechanisms to maintain nutrient supplies. Here we demonstrate that under conditions of dietary restriction or growth factor starvation, where PI3K/mTOR signalling is decreased, matrix-attached human mammary epithelial cells upregulate and internalize β4-integrin along with its matrix substrate, laminin. Endocytosed laminin localizes to lysosomes, results in increased intracellular levels of essential amino acids and enhanced mTORC1 signalling, preventing cell death. Moreover, we show that starved human fibroblasts secrete matrix proteins that maintain the growth of starved mammary epithelial cells contingent upon epithelial cell β4-integrin expression. Our study identifies a crosstalk between stromal fibroblasts and epithelial cells under starvation that could be exploited therapeutically to target tumours resistant to PI3K/mTOR inhibition.

## Abstract (web summary)

Inhibition of PI3K/mTOR, which mimics nutrient starvation, causes death of detached but not matrix-attached cancer cells. Here the authors show that nutrient restriction of epithelial cells causes uptake of the matrix protein laminin, which results in increased intracellular amino acids and enhanced mTORC1 signalling.

PI3K and mTOR signalling plays a key role in mediating cellular responses to growth factor and nutrient availability^1^,^2^. In particular, PI3K activation endows tumours with resistance to dietary restriction^3^. Moreover, it overcomes the cellular requirement for extracellular matrix (ECM) adhesion, rendering the cells anchorage-independent^4^,^5^,^6^,^7^ by preventing metabolic impairment and cell death^8^. Interestingly, our previous studies of breast and ovarian cancer cells showed that pharmacological inhibition of PI3K/mTOR results in the specific apoptosis of matrix-detached tumour cells, whereas ECM-attached cells remain viable. These ECM-attached cells induce an adaptive response, leading to the induction of several pro-survival proteins, including receptor tyrosine kinases, such as IGF1R, EGFR and anti-apoptotic proteins, including Bcl-2 and Bcl-xL^9^. This adaptive response closely mimics the conserved stress responses observed in lower eukaryotes under nutrient deprivation^10^,^11^,^12^,^13^. Intriguingly, it also results in a significant induction of integrins^9^, the trans-membrane proteins that mediate cellular adhesion. Although integrin signalling is required for the adaptive response to occur^9^, the exact role of integrins and matrix adhesion in mediating cell survival in response to PI3K/mTOR inhibition, which mimics starvation, remains unknown. Here we investigate the role of integrins and matrix adhesion in maintaining the survival and homeostasis of mammary epithelial cells under dietary restriction or growth factor-limiting conditions, where PI3K/mTOR signalling is decreased. We find that *in vivo*, mammary fat pads of dietary-restricted (DR) mice have increased β4-integrin expression and enhanced internalization of its substrate, the matrix protein laminin. Consistently, starved mammary epithelial cells in culture internalize laminin along with β4-integrin. Internalized laminin localizes to lysosomes, leading to an increase in intracellular amino acid levels and enhanced mTORC1 signalling, thus preventing cell death. Furthermore, we identify a starvation-induced cellular crosstalk between human epithelial cells and fibroblasts, where laminin-enriched fibroblast-conditioned medium promotes epithelial cell survival, contingent upon expression of epithelial β4-integrin. These findings provide a therapeutic opportunity for targeting cancers resistant to PI3K/mTOR inhibition.

## Results

### Starved epithelial cells internalize β4-integrin

**Figure 1:**
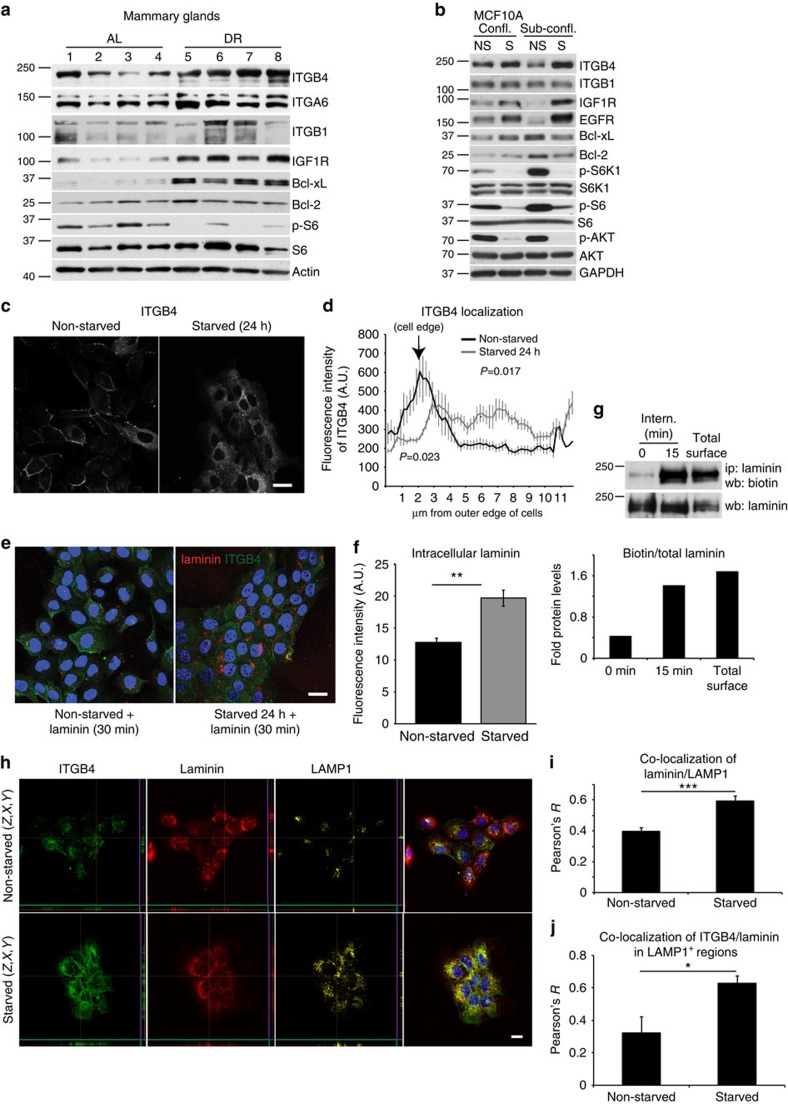
Starved mammary epithelial cells induce β4-integrin expression and internalize laminin. (**a**) Levels of total proteins and S6 phosphorylation (S235/236) in lysates from mouse mammary glands of AL and DR mice (*n*=4). (**b**) Levels of total proteins, phosphorylated S6K1 (T389), S6 (S235/236) and AKT (S473) in lysates from non-starved (NS), 24-h starved (S), confluent (confl.) or subconfluent (sub-confl.) MCF10A cells; GAPDH, glyceraldehyde 3-phosphate dehydrogenase. (**c**,**d**) Confocal images of immunofluorescently stained ITGB4 in non-starved or starved MCF10A cells (**c**), with line scan quantification (**d**) of integrin localization from cell edge towards cell interior, representing the fluorescence intensity average (38 cells per condition); *P* values were measured by Student's *t*-test along the length of the line scan (0.017), or at the cell edge, indicated by the arrow (0.023); scale bar, 30 μm. (**e**,**f**) Confocal images (**e**) and quantification of fluorescence intensity (**f**) of laminin-Alexa-647 (red) added (2.5 μg ml^−1^, 30 min) to non-starved or starved MCF10A cells. The cells were immunostained for ITGB4 (green), and the nuclei counterstained with DAPI (blue); intracellular laminin fluorescence intensity was measured in 28 cells per condition using the confocal mid-sections by ImageJ; Student's *t*-test, **P<0.01; scale bar, 30 μm. (**g**) Top, laminin internalization (intern.) assay in starved MCF10A cells demonstrating increased uptake of biotinylated laminin at the 15 min (compared with 0 min) time point. Total surface bitotinylation is shown as positive control. The cells were lysed and probed for biotin by western blot (wb) after laminin immunoprecipitation (ip). The blot was then stripped and re-probed for total laminin as a loading control; bottom, quantification of biotinylated laminin normalized to total immunoprecipitated laminin. (**h**) Confocal images of non-starved or 24-h starved MCF10A cells that were fed laminin (2.5 μg ml^−1^, 1 h). The cells were stained for ITGB4 (green), laminin (red) or LAMP1 (yellow). *z, x* and *y* planes are shown from images reconstructed from serial sections obtained throughout whole cells and demonstrate intracellular staining; scale bar, 20 μm. (**i**,**j**) Co-localization of laminin and LAMP1 (**i**) or ITGB4 and laminin in LAMP1-positive regions (**j**) as analysed in 150 cells per condition, using ImageJ; Student's *t*-test, *P<0.05 and ***P<0.001. Data in (**d**,**f**,**i**,**j**) represent the mean±s.e.m.

Eight-week-old female Bl6.129 mice were either fed *ad libitum* (AL) a standard rodent diet, or were DR for 18 days. All DR mice received daily meals limiting their total caloric intake to 60% of that of their AL counterparts^3^. The mammary glands were then harvested and the levels of pro-survival proteins examined by western blotting. Interestingly, compared with mammary glands of AL mice, those from DR mice displayed robust induction of the receptor tyrosine kinases, IGF1R and EGFR, as well as the anti-apoptotic protein Bcl-xL (Fig. 1a and Supplementary Fig. 1a), reminiscent of the adaptive response observed in breast and ovarian cancer cells treated with the PI3K/mTOR inhibitor BEZ235 (ref. 9). Although the cancer cells displayed increased expression of either β1-integrin (ITGB1) or β4-integrin (ITGB4) upon BEZ235 treatment^9^ (Supplementary Fig. 1b), only a modest and inconsistent increase in ITGB1 was observed in the mammary glands of DR mice. Nevertheless, a robust increase in ITGB4 and α6-integrin (ITGA6) was noted (Fig. 1a and Supplementary Fig. 1a). To gain mechanistic insight into integrin induction upon dietary restriction, non-transformed MCF10A mammary epithelial cells were used as an *in vitro* culture system, and were subjected to a starvation protocol, thereafter simply referred to as ‘starvation', that deprived them simultaneously of serum and growth factors (EGF, insulin) for 24 h (Supplementary Table 1). This starvation protocol resulted in decreased uptake of nutrients, including glucose and glutamine from the media (Supplementary Fig. 1c), as well decreased Akt activity (Fig. 1b), reminiscent of decreased PI3K signalling and glucose uptake upon matrix detachment^8^. Importantly, this protocol induced an adaptive response in the MCF10A cells that closely mimics the one observed in mammary glands of DR mice *in vivo*. Indeed, *EGFR*, *IGF1R* and *ITGB4* were all induced after a 24-h starvation, at both the protein and mRNA levels, in confluent and subconfluent cellular conditions (Fig. 1b and Supplementary Fig. 1d). Although *ITGB1* expression was slightly elevated at the mRNA level under subconfluent conditions (Supplementary Fig. 1d), its protein levels remained unchanged (Fig. 1b), consistent with the results obtained in the DR mammary glands *in vivo*.

The induction of *ITGB4* under starved conditions prompted us to investigate its functional significance. We first examined whether starvation led to increased localization of ITGB4 to the plasma membrane, where it could potentially contribute to survival through enhanced adhesion signalling^14^. Surprisingly, however, immunofluorescence staining revealed a significant increase in its intracellular localization upon 24-h starvation (Fig. 1c,d). To investigate whether the intracellular integrin was functional in binding laminin, the primary matrix substrate for the ITGB4/A6 heterodimer, fluorescently labelled exogenous laminin (2.5 μg ml^−1^) was added for 30 min to the starved and non-starved MCF10As, followed by fixation and immunostaining for ITGB4. We observed a significant increase in the uptake of fluorescently labelled laminin in the starved cells, suggesting that the ITGB4 is functional in binding matrix proteins, which are then internalized at a higher rate under starved conditions (Fig. 1e,f). Using a biochemical internalization assay where laminin is immunoprecipitated from cell lysates following reversible biotinylation of surface and extracellular proteins, we observed that the extracellular biotinylated laminin is indeed rapidly internalized in starved MCF10A cells (Fig. 1g). Because the localization pattern of the internalized laminin resembled that of lysosomes, we treated starved and non-starved MCF10A cells with exogenous laminin for 1 h and then immunostained the cells for ITGB4, laminin and the lysosomal marker LAMP1. Confocal microscopy demonstrated increased co-localization of laminin with LAMP1, as well as enhanced co-localization of ITGB4 with laminin in LAMP1-positive regions in starved, compared with non-starved cells (Fig. 1h–j). Furthermore, live imaging of starved MCF10A cells expressing RFP-LAMP1 demonstrated rapid internalization of the fluorescently labelled laminin into lysosomes (Supplementary Video 1).

### Laminin uptake enhances mTORC1 activity in starved cells

**Figure 2:**
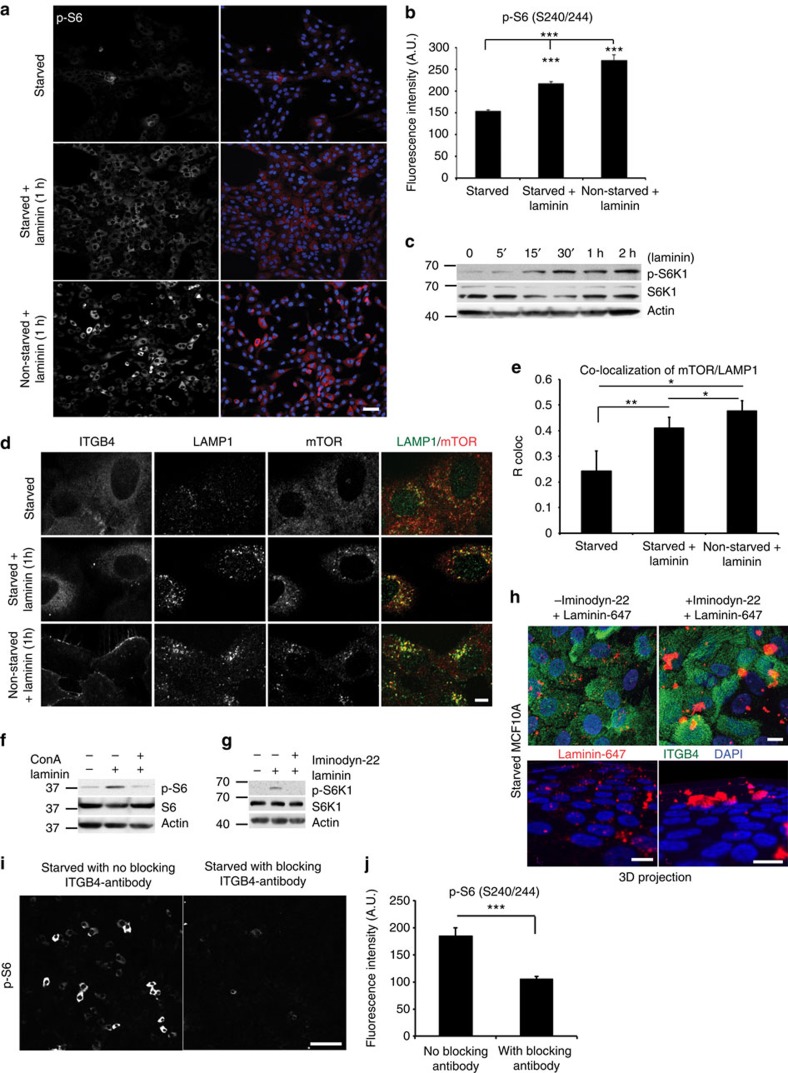
Treatment of starved mammary epithelial cells with laminin results in mTORC1 activation. (**a**,**b**) Confocal images (**a**) and quantification of immunofluorescence (**b**) of p-S6 (S240/244, red) in 24-h starved MCF10A cells, or starved/non-starved MCF10A cells treated with laminin (2.5 μg ml^−1^, 1 h); nuclei were counterstained with DAPI (blue); scale bar, 50 μm. Fluorescence intensity in ∼300 cells per condition was quantified by ImageJ; one-way analysis of variance (ANOVA), Tukey test, ***P<0.001. (**c**) Levels of total proteins and S6K1 phosphorylation (T389) in lysates of 24-h starved MCF10A cells treated with laminin (2.5 μg ml^−1^) for the indicated times. (**d**,**e**) Confocal images (**d**) of MCF10A cells treated as in **a**, showing localization by immunofluorescent staining, of ITGB4, LAMP1 (green), mTOR (red) and overlay of LAMP1 and mTOR; scale bar, 5 μm; co-localization (**e**) was analysed and quantified from confocal sections in ∼50 cells per condition using ImageJ; one-way ANOVA, Tukey test, *P=0.05; **P<0.01. (**f**) Levels of total proteins and phosphorylated S6 (S240/244) in lysates of MCF10A cells treated as in **a**. Where indicated, the cells were first incubated for 10 min with concanamycin A (8 μM), followed by laminin/concanamycin A co-incubation for 1 h and then lysed. DMSO was used as a vehicle control. (**g**) Levels of total proteins and phosphorylated S6K1 (T389) in lysates of MCF10A cells treated as in **a**. Where indicated, the cells were treated for 30 min with the dynamin inhibitor Iminodyn-22 (2 μM), followed by laminin/Iminodyn-22 co-incubation for 1 h. (**h**) Top, confocal images of 24-h-starved MCF10A cells treated with iminodyn-22 for 30 min, before addition of Alexa-647-laminin (red, 2.5 μg ml^−1^, 1 h). The cells were fixed and immunostained for ITGB4 (green) and nuclei counterstained with DAPI (blue). Bottom, three-dimensional projection of cells in the top panel imaged with confocal sectioning, that shows accumulation of extracellular laminin post-iminodyn treatment; scale bar, 10 μm. (**i**,**j**) Confocal images of 24-h starved MCF10A cells incubated with ITGB4-blocking antibody for 3 h, before laminin addition (2.5 μg ml^−1^, 1 h). The cells were immunostained for phosphorylated S6 (S240/244; **i**) and fluorescence intensity-quantified (**j**) as described in **b**; scale bar, 100 μm; Student's *t*-test, ***P<0.001. In **b**,**e**,**j**, data represent the mean±s.e.m.

We next tested whether the addition of exogenous laminin to starved MCF10A cells leads to increased mTORC1 activity. Following a 24-h starvation, MCF10A cells were supplemented with either vehicle control or purified laminin for 1 h. Laminin treatment led to a significant increase (45%) in the phosphorylation of the ribosomal subunit S6, indicating enhanced mTORC1 signalling (Fig. 2a,b). Analysis of the kinetics of mTORC1 activation upon laminin addition revealed a slight increase in phosphorylation of the mTORC1 substrate S6 kinase (S6K1) 15 min following laminin treatment, with maximal phosphorylation occurring at the 30 min time point and sustained thereafter for a total period of at least 2 h (Fig. 2c). Moreover, mTOR localization to lysosomes^15^,^16^,^17^, which occurs in response to amino acid availability, was significantly increased following laminin addition (Fig. 2d,e). Furthermore, treatment of starved MCF10A cells with concanamycin A, which inhibits V-ATPase activity that is required for amino acid sensing and mTORC1 activation^18^, suppressed the increase in S6 phosphorylation upon laminin addition (Fig. 2f).

Receptor-mediated endocytosis is required for the internalization of integrins when bound to their matrix substrates^19^,^20^,^21^. We therefore reasoned that inhibition of endocytosis with a dynamin inhibitor would suppress mTORC1 activation in the starved cells. Indeed, co-incubation of laminin with Iminodyn-22 blocked mTORC1 activation by laminin, as demonstrated by the decreased phosphorylation of its target S6K1 (Fig. 2g). Iminodyn-22 also significantly suppressed the internalization of fluorescently labelled laminin into the starved cells, resulting in significant extracellular laminin accumulation (Fig. 2h).

To assess whether specific binding of laminin to ITGB4 is required for mTORC1 activation, the starved cells were pre-incubated with a ligand-blocking antibody for ITGB4. Upon laminin addition, S6 phosphorylation was suppressed by the antibody treatment, suggesting that increased mTORC1 signalling is dependent on ITGB4-laminin binding (Fig. 2i,j).

### Laminin uptake prevents cell death under starvation

**Figure 3:**
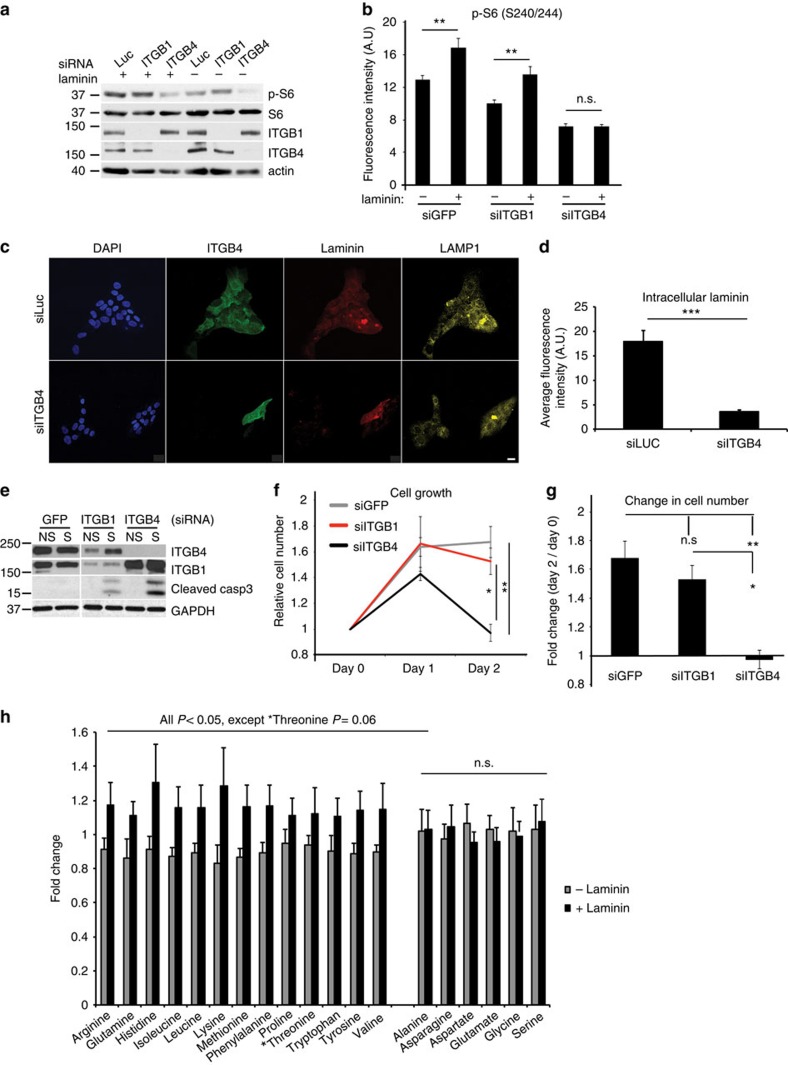
β4-integrin is required for laminin uptake and survival of starved epithelial cells. (**a**) Levels of total proteins and phosphorylated S6 (S235/236) in lysates of MCF10A cells treated with siRNAs for Luciferase (Luc), *ITGB1* or *ITGB4*, and then starved for 24 h and either fed vehicle (PBS) or laminin (2.5 μg ml^−1^) for 1 h. (**b**) MCF10A cells treated as in **a** were immunostained for phosphorylated S6 (S240/244), imaged and fluorescence intensity-quantified using ImageJ (∼400 cells per condition); Student's *t*-test, **P<0.01; n.s., nonsignificant. (**c**,**d**) Confocal images (**c**) of MCF10A cells treated as in **b**, but immunostained for ITGB4 (green), laminin (red) or LAMP1 (yellow); nuclei were counterstained with DAPI (blue); scale bar, 20 μm. Intracellular laminin fluorescence intensity (**d**) was quantified by ImageJ as in **b**, ∼50 cells per condition; Student's *t*-test, ***P<0.001. (**e**) Levels of total proteins in lysates from MCF10A cells treated with siRNAs for *GFP*, *ITGB1* or *ITGB4* and then either non-starved (NS) or starved (S) for 48 h. (**f**,**g**) Relative number of MCF10A cells treated with siRNAs as in **e**, and then starved for 48 h, and counted on days 0, 1 and 2 of starvation (**f**); cell numbers were normalized to day 0 (**g**); *n*=3; one-way ANOVA, Tukey test, *P<0.05; **P<0.01; n.s., nonsignificant. (**h**) Fold changes of amino acid levels in MCF10A cells starved for 24 h and then treated for 1 h with laminin compared with vehicle control. Each value represents the average of four replicates per condition, normalized to the median for each metabolite, with error bars representing s.d.; *P* values were measured by Student's *t*-test. In **b**,**d**,**g**, data represent the mean±s.e.m.

To further assess whether mTORC1 activation is dependent on *ITGB4* expression, either *ITGB1* or *ITGB4* was knocked down by short interfering RNAs (siRNAs; Fig. 3a–d). Compared with control luciferase or *GFP* knockdown, decreased *ITGB1* expression only mildly affected S6 phosphorylation in the starved cells regardless of laminin addition. In contrast, *ITGB4* knockdown resulted in a significant decrease in S6 phosphorylation in the absence of exogenous laminin (Fig. 3a,b). This effect is not surprising, given that laminin is an endogenous component of basement membrane naturally deposited by the epithelial cells. Importantly, *ITGB4* knockdown also suppressed the exogenous laminin-induced increase in S6 phosphorylation observed in the control cells (Fig. 3a,b). This was accompanied by a significant decrease in laminin internalization, compared with cells with either *ITGB1* or luciferase knockdown (Fig. 3c,d). Altogether, these results suggest that ITGB4 is required for laminin internalization and laminin-mediated mTORC1 activation in the starved cells.

To assess whether *ITGB4* expression provides a survival advantage to cells under starved conditions, MCF10A cultures were assayed for cell death and changes in cell number upon integrin knockdown. Western blotting analysis showed that upon starvation, caspase-3 cleavage is highest in *ITGB4* knockdown cells compared with control and *ITGB1* knockdown cells (Fig. 3e). Furthermore, whereas control and *ITGB1* knockdown cells maintained a 1.6-fold increase in cell number 2 days post starvation, *ITGB4* knockdown cells significantly decreased in number, reaching levels quantified on day 0 before starvation (Fig. 3f,g).

Because mTORC1 was activated and localized to lysosomes upon laminin treatment (Fig. 2), we reasoned that the exogenous laminin might lead to increased intracellular amino acid levels in the starved MCF10A cells. In order to test this, we performed liquid chromatography-tandem mass spectrometry (LC-MS) and found that, indeed, the intracellular levels of many amino acids were markedly increased in starved cells upon 1-h treatment with laminin (∼1.3-fold, Fig. 3h). Notably, there were significant increases in the levels of all essential amino acids (EAAs), that is, histidine, isoleucine, leucine, lysine, methionine, phenylalanine, threonine, tryptophan and valine, in addition to glutamine and arginine. Altered amino acid levels were not observed in the control media supplemented with laminin and incubated in the absence of cells, confirming that the purified laminin was not contaminated with free amino acids (Supplementary Fig. 2). These results implied that laminin addition leads to increased intracellular amino acid levels that could be derived from its degradation in lysosomes where it localizes post internalization (Fig. 1e–j and Supplementary Video 1).

Because the starvation medium we used contains amino acids, we deprived the cells of serum and growth factors for 24 h, and of all amino acids for 1 h, and then treated them for an additional hour with laminin. Similar to what we observed in cells treated with starvation medium in the presence of amino acids (Fig. 2), laminin treatment of starved MCF10A cells in the absence of amino acids led to enhanced mTOR localization to lysosomes (Supplementary Fig. 3a,b). This was accompanied by increased S6 phosphorylation that was in turn suppressed by concanamycin treatment (Supplementary Fig. 3c–e). These results indicate that laminin serves as a source of amino acids to the starved MCF10A cells, resulting in enhanced mTORC1 signalling.

To further assess whether the effects of the starvation protocol used in our experiments recapitulate those observed upon dietary restriction *in vivo*, we formulated *in vitro* AL and DR media (Supplementary Table 1). The ‘AL medium' is similar to the ‘non-starvation medium', except that the serum is substituted with 0.9% fatty-acid-free, dialysed albumin, and insulin levels are decreased tenfold to 1 μg ml^−1^. Compared with the ‘AL medium', the ‘DR medium' lacks EGF and contains minimal insulin levels (1 ng ml^−1^). Moreover, the levels of glucose and amino acids in the ‘DR medium' are altered, so as to mimic the fold changes quantified in the plasma of DR compared with AL mice (Supplementary Fig. 4a,b).

We then treated MCF10A cells with the different media (Supplementary Table 1) for 24 h and compared the effects on integrin internalization and mTORC1/AKT signalling. Although the DR medium did not induce ITGB4 expression (Supplementary Fig. 4d), it caused a notable increase in ITGB4 internalization, similar to the effects of the ‘starvation medium' (Supplementary Fig. 4c and Fig. 1c–j). Also similar to the ‘starvation medium', the ‘DR medium' caused significant upregulation of IGF1R and EGFR, along with suppressed mTORC1 and AKT activation (Supplementary Fig. 4d, Fig. 1b and Supplementary Fig. 1a). Importantly, addition of laminin-5 (2.5 μg) for 1 h partially rescued the survival signalling in the ‘DR medium'-treated cells (Supplementary Fig. 4d), reminiscent of what we had observed in cells treated with the ‘starvation medium' (Fig. 2a,b; Fig. 3a,b and Supplementary Fig. 3).

### Fibroblast-secreted matrix promotes epithelial cell survival

**Figure 4:**
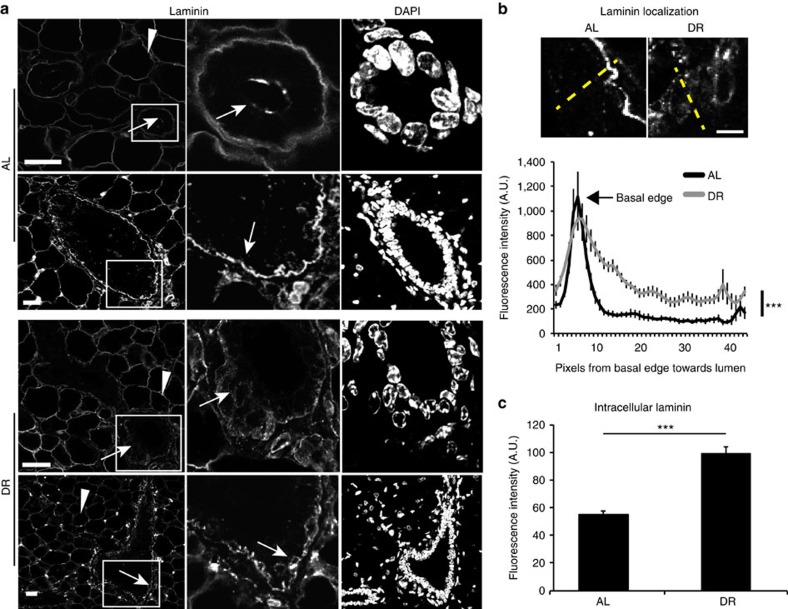
Mammary glands display increased intracellular laminin upon dietary restriction. (**a**) Confocal images of mouse mammary glands isolated from AL or DR mice and immunostained for laminin; arrowheads depict adipocytes and arrows point to mammary glands; scale bars, 10 μm. (**b**) Laminin fluorescence intensity over the axis of whole cells from basal edge to lumen was measured in linescans in Fiji (∼250 cells in ≥10 mammary glands, five mice per group) as marked in yellow (top). The graph (bottom) shows more diffuse laminin staining in the DR conditions with a less well-defined basal edge; Student's *t*-test, ***P<0.001; scale bar, 20 μm. (**c**) Laminin fluorescence intensity was analysed by ImageJ from intracellular regions in ∼120–200 cells from 15 mammary glands, four mice per condition; each value represents the mean±s.e.m.; Student's *t*-test, ***P<0.001.

**Figure 5:**
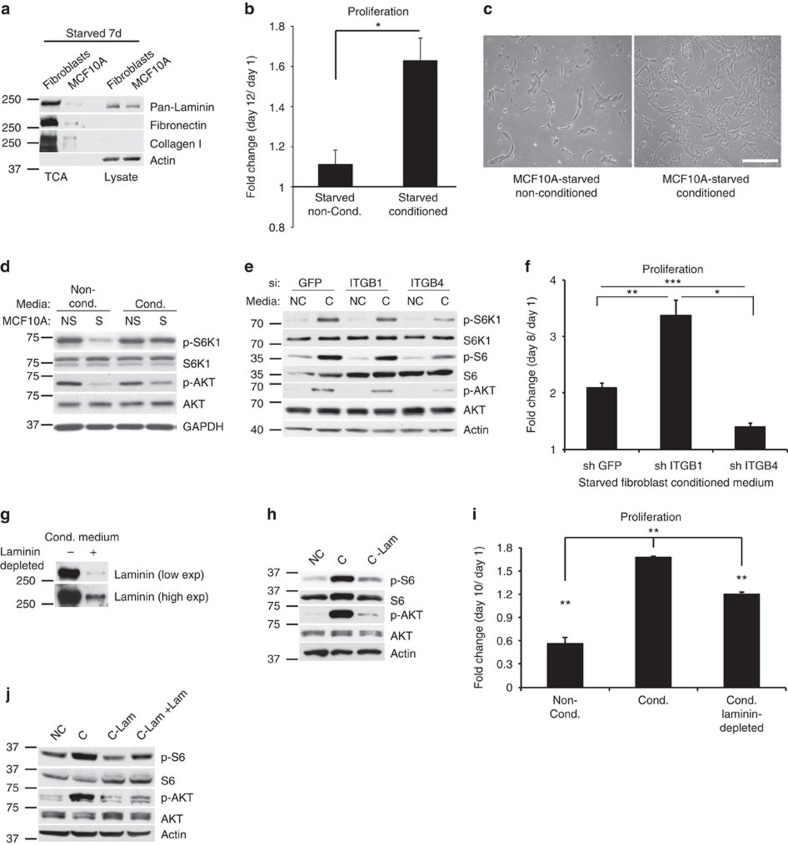
Fibroblasts secrete matrix proteins that enhance epithelial cell survival under starvation. (**a**) Levels of matrix proteins in total cell lysates and TCA-precipitated secreted proteins in the media of primary human fibroblasts or MCF10A cells starved for 7 days. Loading was normalized to cell number. (**b**) Fold proliferation of MCF10A cells that were first starved for 24 h and then treated daily with non-conditioned, or conditioned medium from starved primary human fibroblasts for 12 days; *n*=3; Student's *t*-test, *P<0.05. (**c**) Phase contrast image of MCF10A cells treated as in **b** for 48 h; scale bar, 100 μm. (**d**) Levels of total proteins, phosphorylated S6K1 (T389) and AKT (S473) in lysates from 24-h starved MCF10A cells treated for 1 h with conditioned or non-conditioned media of starved (S) or non-starved (NS) fibroblasts. (**e**) Levels of proteins as in **d** and phosphorylated S6 (S235/236) from MCF10A cells treated with siRNAs for *GFP*, *ITGB1* or *ITGB4*, starved for 24 h, and then incubated with conditioned (C) or non-conditioned (NC) starved fibroblast media. (**f**) Fold proliferation of MCF10A cells with stable knockdown of *GFP*, *ITGB1* or *ITGB4*. The cells were starved for 24 h, and then fed conditioned starved fibroblast medium every 24 h for 8 days; *n*=3; one-way ANOVA, Tukey test, *P<0.05; **P=0.01; ***P<0.001. (**g**) Total laminin levels in TCA-precipitated proteins in the media of 24-h starved primary human fibroblasts, before or following laminin depletion using a laminin antibody affinity column. Loading was normalized to cell number. (**h**) Levels of proteins as in **e** in lysates from 24-h starved MCF10A cells treated for 1 h with NC, C or conditioned laminin-depleted (C-Lam)-starved fibroblast media. (**i**) Fold change in cell number of 24-h starved MCF10A cells fed daily with fibroblast media as in **h** for 10 days; *n*=3; one-way ANOVA, Tukey test, **P<0.01. (**j**) Levels of proteins in MCF10A cells treated as in **h**, or treated with laminin-5 (2.5 μg, C-lam+lam) for 1 h after culture for 24 h in laminin-depleted conditioned medium. In **b**,**f**,**i**, each value represents the mean±s.e.m.

To examine whether intracellular laminin can also be observed *in vivo*, we performed immunofluorescence analysis on mammary gland sections of AL and DR mice. Whereas laminin was mostly localized at the basement membrane under AL conditions, dietary restriction resulted in a notable increase in intracellular laminin, and a decrease in the integrity of basement membrane-localized laminin (Fig. 4a–c). Interestingly, the Coller group has previously shown that contact-inhibited or serum-starved fibroblasts exhibit increased glycolysis, which results in significantly enhanced secretion of ECM proteins^22^,^23^. Consistent with their results, we noted by trichrome staining, which detects matrix proteins, a significant increase in the stroma surrounding the mammary glands under dietary restriction (Supplementary Fig. 5a). We also found that starved primary human fibroblasts display a significant increase in matrix gene mRNA levels (for example, laminin β1 or *LAMB1*, collagen 4A1 or *COL4A1*; Supplementary Fig. 5b). However, mammary epithelial MCF10A cells also showed an increase in *LAMB1* and fibronectin (*FN1*) mRNA levels under starvation (Supplementary Fig. 5c). Nevertheless, trichloroacetic acid (TCA) precipitation of media proteins from either starved fibroblasts or starved MCF10A cells revealed that, although the relative increase at the mRNA level may be similar in both cell types, the fibroblasts contribute significantly more to total secreted matrix proteins, suggesting that fibroblasts are the major matrix-secreting cell type under starved conditions (Fig. 5a). Supplementation of starved MCF10A cells with conditioned media isolated from starved fibroblast cultures significantly increased the growth and survival rates of MCF10A cells (Fig. 5b,c). These results indicate that the fibroblasts secrete factors that contribute to the proliferation and survival of the MCF10A cells under starvation. Furthermore, starved MCF10A cells grown in starved fibroblast-conditioned medium displayed increased survival signalling, particularly S6K1 and AKT phosphorylation, as compared with cells grown in non-conditioned medium (Fig. 5d). This increase was significantly suppressed upon *ITGB4* knockdown (Fig. 5e), suggesting that the pro-survival effect of the fibroblast-conditioned medium is at least partially dependent on *ITGB4* expression. Consistent with these results, knockdown of *ITGB4*, but not *ITGB1*, suppressed starved MCF10A cell proliferation in response to fibroblast-conditioned medium (Fig. 5f).

To assess the contribution of laminin to survival signalling and proliferation, starved MCF10A cells were treated for 1 h with conditioned medium that had first been depleted of laminin by a pan-laminin affinity column (Fig. 5g). This resulted in marked suppression of S6 and AKT phosphorylation (Fig. 5h), as well as significantly decreased proliferation (Fig. 5i), compared with cells treated with control, conditioned medium. However, as expected, this effect was partial, because both the levels of phosphorylated S6 and AKT and the fold increase in cell number were still significantly higher than those of MCF10A treated with non-conditioned control medium (Fig. 5h,i). These results imply that laminin plays a key role in promoting mTORC1 activity and cell survival/growth in the starved epithelial cells, but that other factors secreted in the medium also likely contribute to this effect. Indeed, treatment of the cells with laminin-5 (2.5 μg ml^−1^, 1 h, Fig. 5j) partially rescued the effect on S6 and AKT phosphorylation, further underscoring the role of fibroblast-secreted laminin in epithelial cell survival under starved conditions. Taken together, our findings highlight a starvation-induced crosstalk between fibroblast and epithelial cells that maintains epithelial cell survival, contingent upon matrix adhesion.

## Discussion

Recently, *RAS*-transformed cancer cells were shown to uptake extracellular proteins, particularly serum albumin, via an endocytic process termed macropinocytosis^24^,^25^,^26^. The albumin served as a source for glutamine, a major nutrient that can support the cancer cells' metabolic needs. Whereas low levels of glutamine were detected in *KRAS*-driven pancreatic tumours compared with normal adjacent tissue, intratumoral accumulation of EAAs was concomitantly observed and thought to result from the lysosomal degradation of scavenged albumin^25^.

In contrast, our study highlights a distinct process involving matrix internalization by starved normal epithelial cells that is dependent on *ITGB4* expression. We show that laminin, an easily accessible extracellular protein, is endocytosed and internalized into lysosomes, providing a source of EAAs that support starved epithelial cell survival. Although laminin endocytosis has previously been reported^27^, this process involved a distinct receptor and occurred in a different tissue context not linked to metabolism or starvation. Our study shows instead that ITGB4-mediated laminin internalization occurs under conditions of decreased PI3K/mTOR signalling, which mimic loss of nutrient uptake.

Activation of mTORC1 was recently reported to suppress proliferation when the cells rely on extracellular proteins as a source of amino acids^26^. Our study is in line with this finding, as enhanced mTORC1 activity was only observed post internalization of laminin, which could re-set mTORC1 activity to levels closer to those observed under non-starved conditions, in turn preventing excessive matrix protein uptake. Furthermore, our study is consistent with another recent report^28^, demonstrating that nutrient deprivation, which suppresses mTORC1 activity, results in the accumulation of integrins in subnuclear compartments followed by their internalization into lysosomes, a process necessary for lysosomal recruitment and activation of mTOR. Indeed, we show that under conditions of growth factor and serum deprivation, which mimic loss of nutrient uptake and cause decreased mTORC1 activity, laminin-bound integrin internalization is enhanced, in turn leading to mTORC1 re-activation.

In addition, our results highlight a novel role for ECM in providing nutrient supplies to starved epithelial cells. Although the source of matrix could be the epithelial cells themselves, it is very likely that stromal cells, which secrete significant amounts of matrix proteins upon starvation, represent a major ECM protein source for the epithelial cells. Therefore, our study implies a crosstalk between epithelial cells and neighbouring stroma that provides an alternative nutrient source for the epithelial cells under starvation conditions, promoting their survival. Consistently, *in vivo* dietary restriction leads to a significant increase in matrix deposition around the mammary epithelial ducts, and intracellular laminin localization, suggesting that under these conditions, an abundance of matrix proteins is available for the starved epithelial cells to use for survival. These findings provide a potential therapeutic opportunity through targeting of integrins in stromally enriched cancers that are resistant to PI3K/mTOR inhibition, which mimics starvation.

## Methods

### Reagents

All reagents were obtained commercially, except for LAMP1-mRFP-FLAG^X2^ plasmid, which was a kind gift from Dr Roberto Zoncu^18^. Antibodies for AKT (40D4, #2920), p-AKT S473 (D9E #4060), Bcl-xL (54H6, #2764), caspase-3 (8G10, #9665), 4E-BP1 (53H11 #9644), EGFR (D38B1, #4267), α6-integrin (#3750), mTOR (7C10, #2983), S6 (54D2, #2317), p-S6 S240/244 (D68F8 #5364), p-S6 S235/236 (2F9, #4856), S6K1 (49D7, #2708) and p-S6K1 T389 (#9205) are from Cell Signaling Technologies (CST); Collagen 1 (ab292, ab6308) and fibronectin (ab2413) from Abcam (ab); β-actin (AM4302) and β-tubulin (32–2600) from Invitrogen; GAPDH (sc-25778) and LAMP1 (sc-20011) from Santa Cruz (sc); IGF1Rβ (111A9, CST #3018 and sc-713); β1-integrin (BD Biosciences #610468 and EMD Millipore, MAB1959Z and activation-specific MAB2247 clone12G10); β4-integrin (CST #4707 and ab110167); β4-integrin-blocking antibody (Millipore, MAB2059); laminin antibodies (Dako, Z0097, Millipore MAB19562 and Sigma HPA001908); Bcl-2 (EP36, Epitomics #AC-0035). Antibodies for western blotting were diluted at a ratio of (1:1,000), except for p-S6K1 (1:500), Bcl-xL and p-S6 (1:3,000), actin and tubulin (1:20,000). All antibodies for immunofluorescence were diluted at a ratio of 1:100.

Laminin was obtained from Sigma (#L2020); laminin-5 from Millipore (CC145) and Kerafast (EUV101); BEZ235 from Axon Medchem^29^; Iminodyn-22 (ab120461) from Abcam; Concanamycin A (#C9705) from Sigma. Laminin (Sigma #L2020) was fluorescently labelled with Alexa-647 using a protein-labelling kit (Molecular Probes #A-20173) and illustra Nap-5 Columns (GE Healthcare 17-0853-01). ON-TARGET Plus SMARTpool siRNAs targeting Luciferase, *GFP*, human *ITGB1* (#L-004506-00-0005) and human *ITGB4* (#L-008011-00-0005) were purchased from Dharmacon and transfected according to the manufacturer's protocol.

Short hairpin RNAs for *ITGB4* and control sh*GFP* were obtained from the RNAi Consortium; pLKO sh*ITGB4*: TRCN0000057768-72; control pLKO.1 sh*GFP* (CTACAACAGCCACAACGTCCT). Short hairpin RNA for *ITGB1* was obtained from Open Biosystems pTRIPZ ITGB1 V2THS-133469 (Open Biosystems). ITGB4-blocking antibody (10 μg ml^−1^) was added to starved MCF10A cells 3 h before addition of laminin for 1 h.

### Cell culture

All cells were maintained at 37 °C in a humidified incubator with 5% CO_2_ and were tested negative for mycoplasma contamination. The breast cancer cell line MCF7 and the ovarian cancer cell lines MCAS and OV2008 were a kind gift from Dr D. Slamon (UCLA), and were authenticated by STR sequencing. MCF7 cells were cultured in DMEM, MCAS and OV2008 in a 1:1 mixture of MCDB105 (Cell Applications Inc.) and 199 media (Invitrogen). Media for MCF7, OV2008 and MCAS were supplemented with 10% inactivated calf serum, 2 mM L-glutamine and 1:100 penicillin–streptomycin (Invitrogen). Non-transformed mammary epithelial MCF10A cells were obtained from the Karmanos Cancer Institute and cultured in ‘non-starvation' growth medium, as previously described^30^: DMEM-F12 was supplemented with 5% horse serum (Invitrogen), 20 ng ml^−1^ EGF (Peprotech), 0.5 μg ml^−1^ hydrocortisone, 100 ng ml^−1^ cholera toxin, 10 μg ml^−1^ insulin (Sigma) and penicillin–streptomycin (Invitrogen). The ‘starvation medium' used in all experiments consists of the ‘non-starvation' medium deprived of serum, EGF and insulin. The ‘AL medium' is similar to the ‘non-starvation medium' except that the serum is substituted with 0.9% fatty-acid-free dialysed albumin, and the insulin levels are decreased to 1 μg ml^−1^. Compared with the ‘AL medium', the ‘DR medium' lacks EGF and contains minimal insulin levels (1 ng ml^−1^). Moreover, the levels of glucose and amino acids in the ‘DR medium' are altered so as to mimic the fold changes quantified in the plasma of DR compared with AL mice (recipes for all media are described in Supplementary Table 1). Breast cancer cells MCF10A.DCIS.com were obtained from the Karmanos Cancer Institute and cultured in the same ‘non-starvation' medium as MCF10A cells. Human primary fibroblasts were purchased from Life Technologies (C0045C) and cultured in Medium 106 (Life Technologies) with low-serum growth supplement (Life Technologies). For conditioned medium experiments, the human fibroblasts were first grown in their ‘starvation medium' consisting of Medium 106 deprived of all supplements for 2–7 days. The medium was then refreshed (with starvation medium) and collected every 24 h to feed the 24-h-starved MCF10A cells. Cell number was counted on the indicated days using a coulter counter. For experiments with Iminodyn-22 the cells were pre-incubated with the inhibitor for 30 min before adding the labelled laminin. For generation of MCF10A cells with stable *ITGB1*, *ITGB4* or control *GFP* knockdown, lentiviral supernatants produced from plasmids encoding the corresponding hairpins were used, and infected cells were selected for at least 7 days with 4 μg ml^−1^ puromycin.

### Amino acid withdrawal and concanamycin A treatment

MCF10A cells were first starved of serum and growth factors for 24 h using the ‘starvation medium' containing amino acids. Then, the medium was replaced with medium deprived of serum, growth factors and all amino acids, and the cells incubated for 1 h before treatment for an additional hour, with 2.5 ug ml^−1^ laminin. For concanamycin treatment experiments, 10 min before addition of laminin, either concanamycin A (8 μM) or vehicle (DMSO) was added to the cells followed by 1 h co-incubation with laminin. The cells were then fixed and processed for immunofluorescence imaging as described below.

### Mouse studies

All animal studies and procedures were approved by the Animal Care and Use Committee at Boston Children's Hospital. Eight-week-old female Bl6/129 svJae mice were subjected to 40% dietary restriction for 18 days, as previously described^3^. The mice were individually caged for at least 4 days before subdividing them into an AL-fed group (Prolab RMH 3000, 5P76) and a dietary restriction group (LabDiet, 5B6V). Weekly body weights and food intake were recorded. At necropsy, the fourth pair of inguinal fat pads was harvested and either fixed in formalin or snap-frozen in liquid nitrogen for later processing.

### Laminin depletion from conditioned media

Starved conditioned fibroblast medium was collected from the cells daily, and passed through an antibody affinity column at 4 °C, at a rate of 5 ml h^−1^. The medium was collected and filtered before adding it to the starved MCF10A cells. The control, conditioned medium was also filtered and kept on ice for the same amount of time. The laminin affinity column was prepared by conjugating 5 mg of pan-laminin antibody (Dako, Z0097) to 2 g of CNBr-activated Sepharose 4B for 24 h at 4 °C.

### Biotin-based laminin internalization assay

The laminin internalization experiment was done as previously described^31^. Briefly, overnight-starved cells were placed on ice, and the extracellular and membrane proteins were biotin-labelled in Hank's balanced salt solution with 0.5 mg ml^−1^ of EZ-Link Sulfo-NHS-SS-Biotin (Life Technology #21328) for 30 min. Unbound biotin was washed with cold PBS (pH 8.0), and the cells were removed from ice and incubated in warm ‘starvation medium'. The biotin-labelled surface proteins were allowed to internalize for 15 min, and the cells were again placed on ice and washed with ice-cold PBS (pH 8.0). The remaining biotin on the surface was removed by incubating for 30 min with 60 mM MesNa (sodium 2-mercaptoethanolsulfate, Sigma #M1511), followed by quenching with 100 mM iodoacetamide (Sigma #I1149). As a control for total surface biotin labelling, one plate of cells was left without MesNa treatment and lysed immediately following biotin labelling. As a control for surface biotin removal, another plate of cells was instead treated with MesNa immediately following biotin labelling and lysed. The cells were lysed as in ref. 31 and the cell lysates cleared by centrifugation. The supernatant was then incubated for 1 h with 1:100 pan-laminin antibody (Dako, Z0097), which had been conjugated with A/G-sepharose beads for 2 h before this incubation. Internalized laminin was detected with horseradish peroxidase (HRP)-conjugated biotin antibody (CST #7075), and total laminin with pan-laminin antibody (Dako, Z0097) followed by incubation with mouse anti-rabbit IgG conformation-specific secondary antibody (CST #5127).

### Immunofluorescence staining and microscopy

For confocal imaging, the cells were grown on No. 1.5 coverslips, fixed with 4% paraformaldehyde and immunostained as follows. Cells were permeabilized with 0.05% Triton X-100 in PBS for 15 min, blocked with 5% goat serum in PBS, and then incubated overnight with primary antibodies (1:200 dilution) at 4 °C. Secondary antibodies were Alexa fluorophore-conjugated (−488, −568, −647, Molecular Probes) and used at 1:400 dilution in 5% goat serum, the nuclei were counterstained with 4,6-diamidino-2-phenylindole (DAPI) for 5 min. Tissue sections were de-paraffinized for 2 × 10 min in xylene and hydrated through ethanol series (100, 90, 70, 50 and 20% H_2_O) for 5 min each. Antigen retrieval was performed by incubating the sections in sodium citrate buffer (pH 6.0) in a steamer for 30 min (p-S6 staining) or, alternatively, by 7-min treatment with proteinase K (laminin staining). The slides were then blocked in 5% goat serum and incubated with primary antibody overnight (1:100), followed by 30-min Alexa-conjugated secondary antibody incubation (1:400). Nuclei were counterstained with DAPI. All confocal images were collected with a Nikon A1R point scanning confocal on a Nikon Ti-E inverted microscope equipped with Plan Apo VC × 20/0.75 numerical aperture (NA), Plan Fluor × 40/1.3 NA and Plan Apo × 60/1.4 NA, and the Perfect Focus System for continuous maintenance of focus. DAPI fluorescence was excited with the 404 nm laser line with a solid-state laser and a 450/50 emission filter. Alexa-488 was excited with 488 nm Argon-krypton laser with a 525/50 emission filter. Alexa-568 was excited with 561 nm solid-state laser with a 595/50 emission filter and the Alexa-647 was excited with a 640 nm solid-state laser with a 700/75 emission filter. Images were acquired with the Nikon Elements acquisition software. For figures on laminin-647 internalization, confocal z-series were taken, 26 optical sections were collected with a step size of 0.125 microns, using a TiZdrive. Z-series are shown as maximum z-projections and were processed by the Nikon Elements software. Light microscopy was used to obtain images of cells in culture as well as trichrome-stained tissue sections.

### Live cell imaging

MCF10A cells were transfected with LAMP1-mRFP-FLAG^X2^, starved for 24 h and then treated with fluorescently labelled laminin-Alexa-647. The cells were imaged with Nikon A1R point scanning confocal microscope, with Perfect Focus System, and a × 60 Plan Apo Oil lens, over a span of 60 min and images were acquired every 10 s. The cells were kept in a humidified, 5% CO_2_-supplemented, 37 °C chamber during imaging.

### Tissue trichrome staining

Mammary gland tissue sections were stained with trichrome at the Rodent Histopathology Core Facility at the Dana-Farber/Harvard Cancer Center. Four individuals separately scored the intensity of collagen (blue) staining, as high or low, in a blinded manner.

### Metabolite extraction and amino acid quantification

For cell lysates, MCF10A cells were plated on a six-well plate (3 × 10^5^ per well) 24 h before they were either non-starved or starved for another 24 h. The cells were then incubated with 2.3 μg ml^−1^ laminin-5 (Kerafast) for 1 h before harvest. Metabolites were harvested by washing with cold PBS, adding 1 ml of ice-cold 80% methanol (LC-MS grade), incubating the cells for 15 min at −80 °C, and scraping the lysates to tubes on dry ice. Cell debris was pelleted by centrifugation (9,000*g*, 4 °C, 10 min). A volume of 100 μl of the resulting supernatant was dried using a nitrogen evaporator and resuspended in 100 μl extraction solution (75% acetonitrile, 25% methanol, 0.2% formic acid). The suspension was centrifuged (10,000*g*, 4 °C, 10 min) and analysed using LC-MS. For plasma, metabolites were extracted by combining 10 μl plasma with 90 μl extraction solution. The suspension was centrifuged (10,000*g*, 4 °C, 10 min) and the supernatant was analysed using LC-MS.

LC-MS data were acquired using a hydrophilic interaction liquid chromatography method with positive ion mode mass spectrometry operated on Nexera X2 UHPLC (Shimadzu Scientific Instruments, Marlborough, MA) coupled to a Q Exactive orbitrap mass spectrometer (Thermo Fisher Scientific, Waltham, MA) as described previously^32^.

### Plasma glucose quantification

Plasma glucose levels were measured using a glucose oxidase kit (Thermo Fisher Scientific).

### Glucose and glutamine uptake assay

MCF10A cells were plated 24 h before the assay. Cells were incubated with ‘non-starvation' or ‘starvation medium', and medium-only samples were incubated simultaneously in the absence of cells. Media were harvested from the cells and from no-cell control wells and spun briefly. The indicated metabolites were then analysed in the supernatant by Nova BioProfiler FLEX (Applitech Pharma), and the results normalized to cell number.

### Quantitative real-time PCR

Total mRNA was isolated with TRIzol (Invitrogen) according to the manufacturer's instructions, treated with DNase I (RNase-free, Roche Molecular Biochemicals) and reverse-transcribed into cDNA with random hexamers using the SuperScript II First-Strand Synthesis System (Invitrogen). Primers listed below were validated and PCR reactions performed as previously described^33^. Quantitative PCR reactions were performed in triplicates using an Applied Biosystems ViiA 7 Real-Time PCR system. PCR reactions contained cDNA resulting from reverse transcription of 25 ng total RNA, 150 nM of each primer and 5 μl 2X-Jump Start SYBR Green PCR Mix (Invitrogen) in 10 μl total volume. Relative mRNA levels were calculated using the comparative C_T_ method and normalized to cyclophilin. The QPCR Primers used are: *COL4A1*: 5′-CCAGGGGTCGGAGAGAAAG-3′ and 5′-GGTCCTGTGCCTATAACAATTCC-3′; CyclophilinA (*CYPA*): 5′-GGAGATGGCACAGGAGGAA-3′ and 5′-GCCCGTAGTGCTTCAGCTT-3′; *EGFR*: 5′-AGGCACGAGTAACAAGCTCAC-3′ and 5′-ATGAGGACATAACCAGCCACC-3′; *FN1*: 5′-CGGTGGCTGTCAGTCAAAG-3′ and 5′-AAACCTCGGCTTCCTCCATAA-3′; *IGF1R*: 5′-AAGGAATGAAGTCTGGCTCCG-3′ and 5′-CAGCTGCTGATAGTCGTTGC-3′; *ITGB1*: 5′-CCTACTTCTGCACGATGTGATG-3′ and 5′-CCTTTGCTACGGTTGGTTACATT-3′; *ITGB4*: 5′-GCTTCACACCTATTTCCCTGTC-3′ and 5′-GACCCAGTCCTCGTCTTCTG-3′; *LAMB1*: 5′-CACAAGCCCGAACCCTACTG-3′ and 5′-GACCACATTTTCAATGAGATGGC-3′.

### Western blotting

Western blot samples were harvested and lysed either directly in reducing SDS sample buffer (Boston BioProducts BP-111R) or RIPA buffer (Tris-HCl 50 mM pH 7.4, NaCl 150 mM, NP-40 1%, sodium deoxycholate 0.5%, SDS 0.1%) supplemented with protease and phosphatase inhibitors (Leupeptin, Aprotinin, 1 mM phenylmethyl sulphonyl fluoride, Pepstatin, 1 mM sodium fluoride, 1 mM sodium orthovanadate). Lysates were clarified by centrifugation at 13,000*g* for 10 min and reducing SDS sample buffer was added. The cleared lysates were then boiled in 1 × sample buffer for 5 min and resolved by 4–20% SDS–PAGE gradient gels (Invitrogen), transferred to polyvinylidene difluoride membranes (Whatman), blocked with 5% BSA in TBS (25 mM Tris, 140 mM NaCl, 2.7 mM KCl, pH 7.4), 0.1% Tween 20 for 1 h at room temperature and probed by antibodies overnight. Membranes were subsequently probed with secondary antibodies linked to HRP (GE Healthcare). Western blot membranes were developed using Luminata Western HRP substrate for detection of HRP (Millipore). Western blot results were prepared using Kodak film developer and Epson 3000 scanner. Scanned films of uncropped blots are shown in Supplementary Figs 6–8.

### TCA protein precipitation

For TCA protein precipitation from the media, cells were cultured at similar confluences, fresh serum-free media were administered to the cells 24 h before the media were collected and the cells counted. Proteins were precipitated from the media by adding ¼ volume of ice-cold TCA and incubated on ice for 25 min. Samples were spun at 18,000*g* for 20 min. Protein pellets were washed twice with ice-cold acetone and spun 18,000 g for 5 min after each wash. Pellets were air-dried and resuspended in SDS sample buffer. Samples were normalized to cell number and used for western blotting.

### Image analysis

Image analysis was performed with the ImageJ 1.48q Fiji analysis software. For fluorescence intensity comparisons, images were acquired at identical settings and intensity was analysed. Intracellular laminin was quantitated from confocal mid-sections of cells with the number of cells analysed separately indicated in each figure. Background was subtracted for the analysis of p-S6 in the blocking antibody experiment, for analysis of intracellular laminin in tissues and for all co-localization analyses. For other intensity quantifications, the background was measured and, if found similar in all conditions, was not subtracted. For linescans, fluorescence intensity was measured over an equal distance from the cell periphery across the whole cell. Co-localization was analysed using the Coloc2 plugin in ImageJ, and either two channels were selected to assess co-localization, or a third image was used to define regions of interest (LAMP-positive areas) to assess co-localization of ITGB4 and laminin in those areas. As an output to measure linear correlation, Pearson's R result is shown from the Coloc2 analysis.

### Statistics

All experiments were repeated at least three independent times. All samples represent biological replicates. No samples or animals were excluded from analysis and sample size estimates were not used. Animals were randomly assigned to groups. Studies were not conducted blinded, except for histological analyses. Data represent the mean±s.e.m, unless otherwise indicated. In comparing two groups, a two-tailed non-paired Student's *t*-test was conducted. For three or more groups, one-way analysis of variance was conducted, followed by a *post hoc* Tukey test. Nonparametric two-tailed Fisher exact test was conducted for analysis of stain intensity scores. *P*≤0.05 was considered significant.

### Data availability

The authors declare that data supporting the findings of this study are available within the paper and its Supplementary Information.

## Additional information

**How to cite this article:** Muranen, T. *et al*. Starved epithelial cells uptake extracellular matrix for survival. *Nat. Commun.* **8,** 13989 doi: 10.1038/ncomms13989 (2017).

**Publisher's note:** Springer Nature remains neutral with regard to jurisdictional claims in published maps and institutional affiliations.

## Supplementary Material

Supplementary Information Supplementary Figures and Supplementary Table 1

Supplementary Movie 1 Exogenous laminin is rapidly internalized into lysosomes of starved mammary epithelial cells.
