# Supplementary material for: Starved epithelial cells uptake extracellular matrix for survival
Source: Nat Commun. 2017 Jan 10;8:13989. doi: 10.1038/ncomms13989 (PMC5234072; doi:10.1038/ncomms13989)
Supplement: Supplementary Information — Supplementary Figures and Supplementary Table 1 [file ncomms13989-s1.pdf]

Supplementary Figure 1

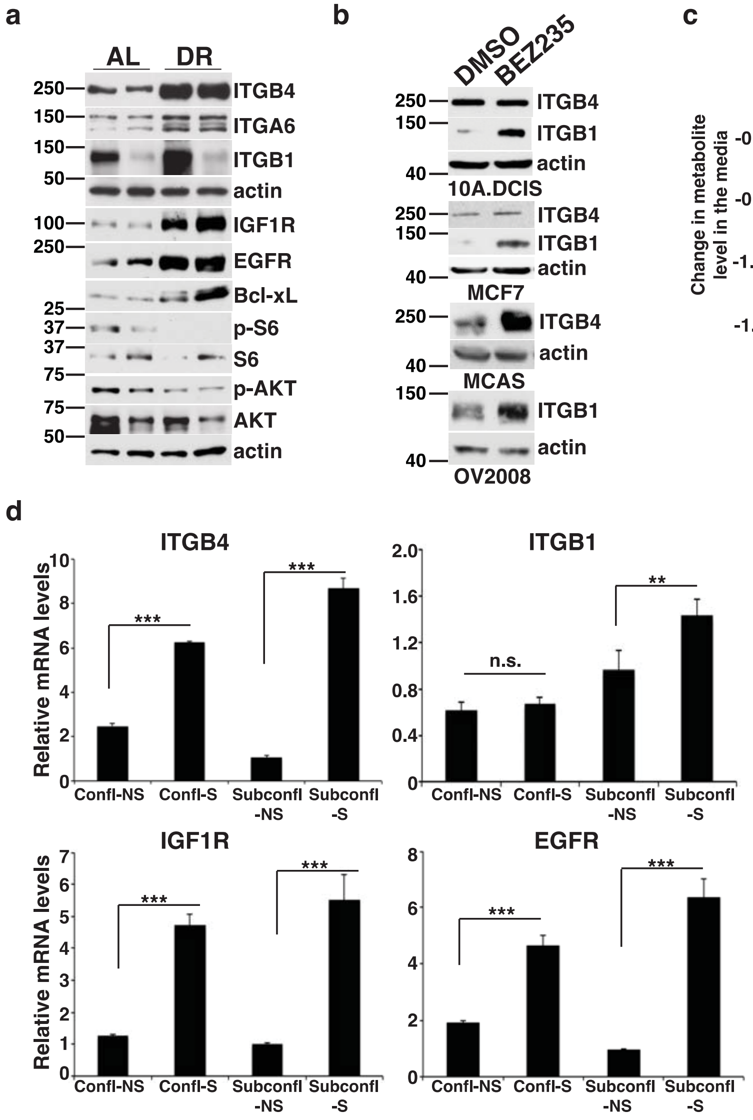

Serum and growth factor deprivation result in decreased nutrient uptake in MCF10A cells and increased β4-integrin expression. (**a**) Levels of total proteins, phosphorylated S6 (S235/236) and Akt (S473) in lysates from mammary glands of *ad libitum*-fed (AL) or dietary-restricted (DR) female mice (n=2). (**b**) Levels of total proteins in lysates from breast (MCF7 and MCF10A.DCIS) and ovarian (MCAS and OV2008) cancer cells treated for 24 hours with the dual PI3K/mTOR inhibitor BEZ235 (0.5μM) or control vehicle dimethyl sulfoxide (DMSO). (**c**) Glucose and glutamine uptake by MCF10A cells that were either non-starved or starved of serum and growth factors for 24 hours. Media metabolite levels as measured by Nova BioProfiler Flex were normalized to cell number and the experiment was performed in triplicates; *p<0.05, **p<0.01. (**d**) QPCR analysis of indicated gene expression in MCF10A cells grown in non-starved (NS) or 24-hour starved (S), confluent or sub-confluent conditions; n=3; **p<0.01, ***p<0.001; n.s., non-significant. In (c) and (d), each value represents the mean ± s.e.m. All p-values were measured by Student's t-test.

Supplementary Figure 2

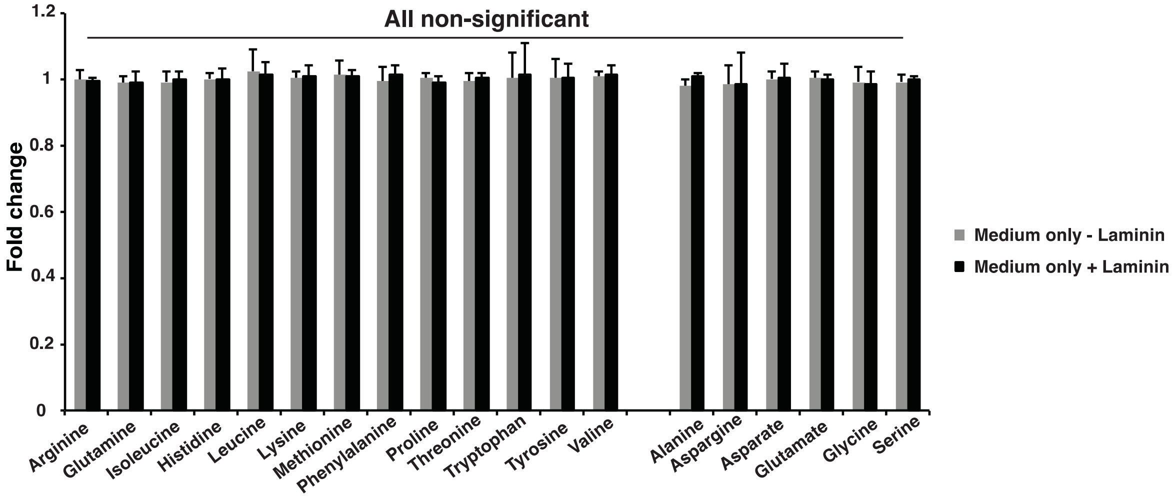

Amino acid levels do not change in cell-free medium incubated with laminin. Relative levels (fold change) of amino acids in MCF10A starvation medium incubated for 24 hours without cells and then supplemented or non-supplemented with laminin (2.3 μg ml^{-1}) for 1 hour. Each value represents the average of 4 replicates per condition, normalized to the median for each metabolite, with error bars representing s.d.; statistical significance was assessed by one-way ANOVA, Tukey test.

Supplementary Figure 3

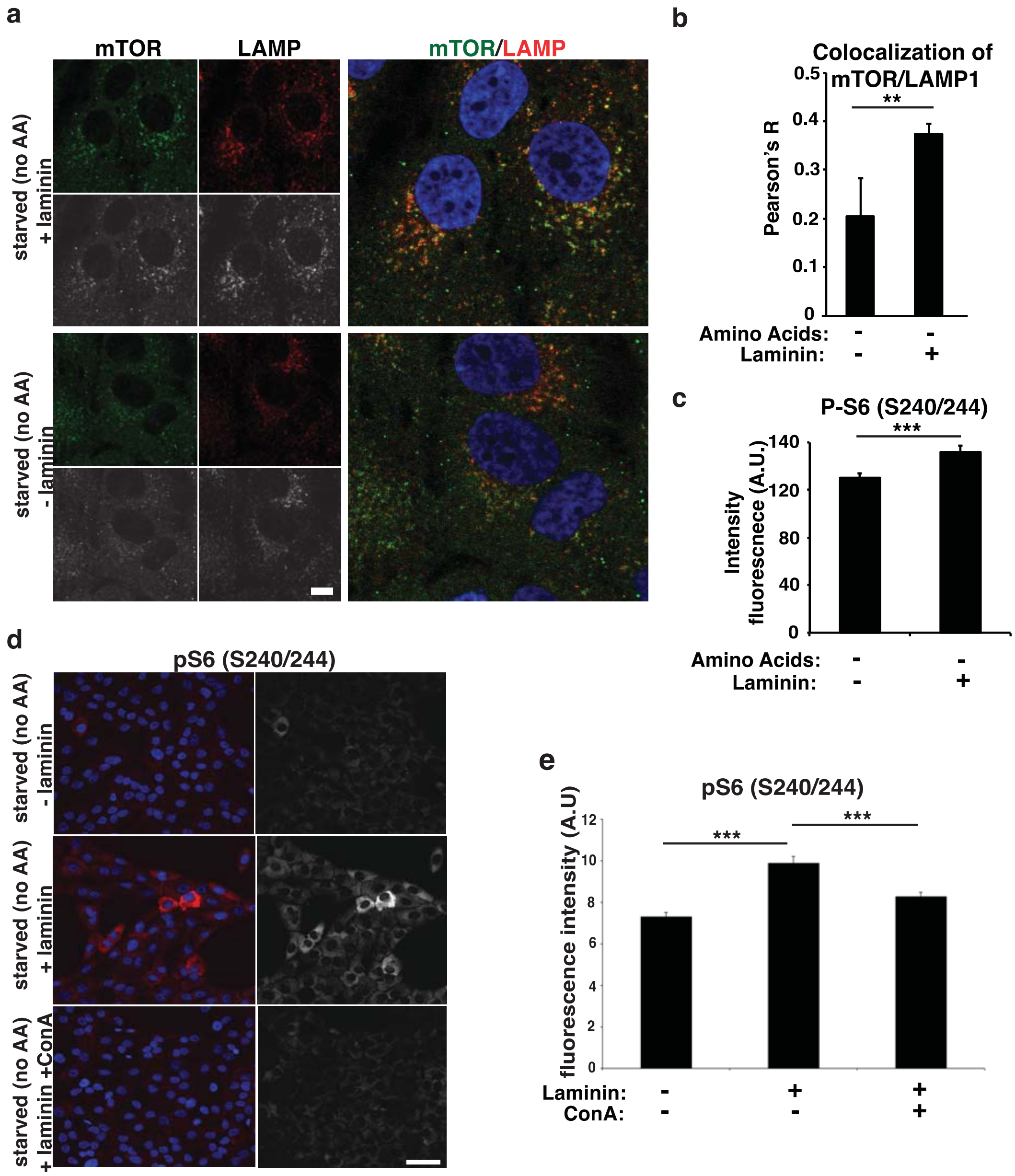

Laminin addition to MCF10A cells incubated in amino acid-free medium increases mTORC1 signalling. (**a**) MCF10A cells were starved of serum and growth factors for 24 hours, and then starved for an additional hour of serum, growth factors and all amino acids. Laminin (2.5 µg ml^{-1}) was then added to the cells for 1 hour and the cells were fixed and immunostained for LAMP1 (red) and mTOR (green); scale bar 10µm . (**b,c**) Co-localization of LAMP1 and mTOR in (a) was analysed by ImageJ and is represented by Pearson's R correlation (**b**); In the same experiment, phospho-S6 (S240/244) was stained and fluorescence intensity analysed with or without laminin addition (**c**); ~100 cells were analysed per condition; Student's t-test, **$p<0.01$, ***$p<0.001$. (**d,e**) MCF10A cells were treated as in (a), concanamycin A (8µM) was added 10 minutes prior to treating the cells with 2.5µg ml^{-1} laminin for 1 hour. The cells were then stained for p-S6 (S240/244, red) and DAPI (blue) and imaged (d); Fluorescence intensity was quantified and analysed from ~200 cells per condition (e); scale bar: 50µm; one-way ANOVA, Tukey test, ***$p<0.001$. In (b), (c), and (e), each value represents the mean ± s.e.m.

Supplementary Figure 4

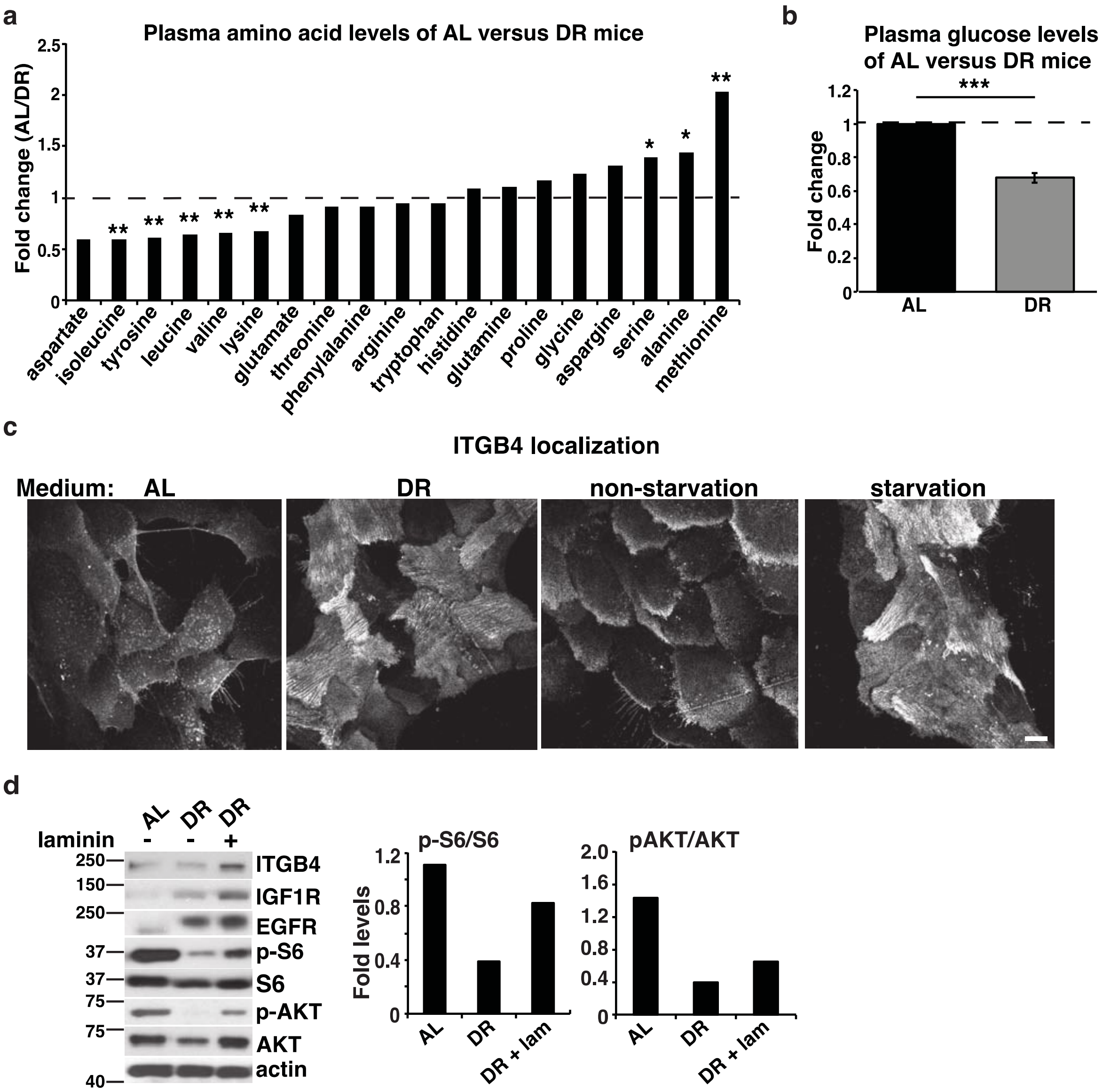

Dietary restriction medium mimics the effects observed with the serum/growth factor starvation medium. (**a**) Fold differences of amino acid levels in the plasma of *ad-libitum*-fed (AL) or dietary restricted (DR) mice, as measured by LC-MS. Each value represents the average of 4 replicates per condition, normalized to the median for each metabolite;one-way ANOVA, Tuket test, *p<0.05, **p<0.01. (**b**) Fold difference of plasma glucose levels in AL versus DR mice as measured by the glucose oxidase kit with error bars representing s.e.m.; Student's t-test, ***p<0.001. (**c**) Confocal images of MCF10A cells that were fed either "AL", "DR", "non-starvation" or "starvation" medium for 24 hours and were immunostained for ITGB4. The images show ITGB4 localization patterns similar for: "AL" versus "non-starvation" media; as well as "DR" versus "starvation" media; scale bar: 5μm. (**d**) Left, levels of total proteins, phosphorylated S6 (S240/244) and AKT (S473) in lysates from MCF10A cells grown for 24 hours in either "AL medium", "DR medium", and either treated or non-treated with laminin-5 (2.5μg ml^{-1}, 1 hour); Right, quantification by ImageJ of phosphorylated S6 and AKT demonstrating partial rescue of survival signalling upon laminin treatment of cells grown in "DR medium".

Supplementary Figure 5

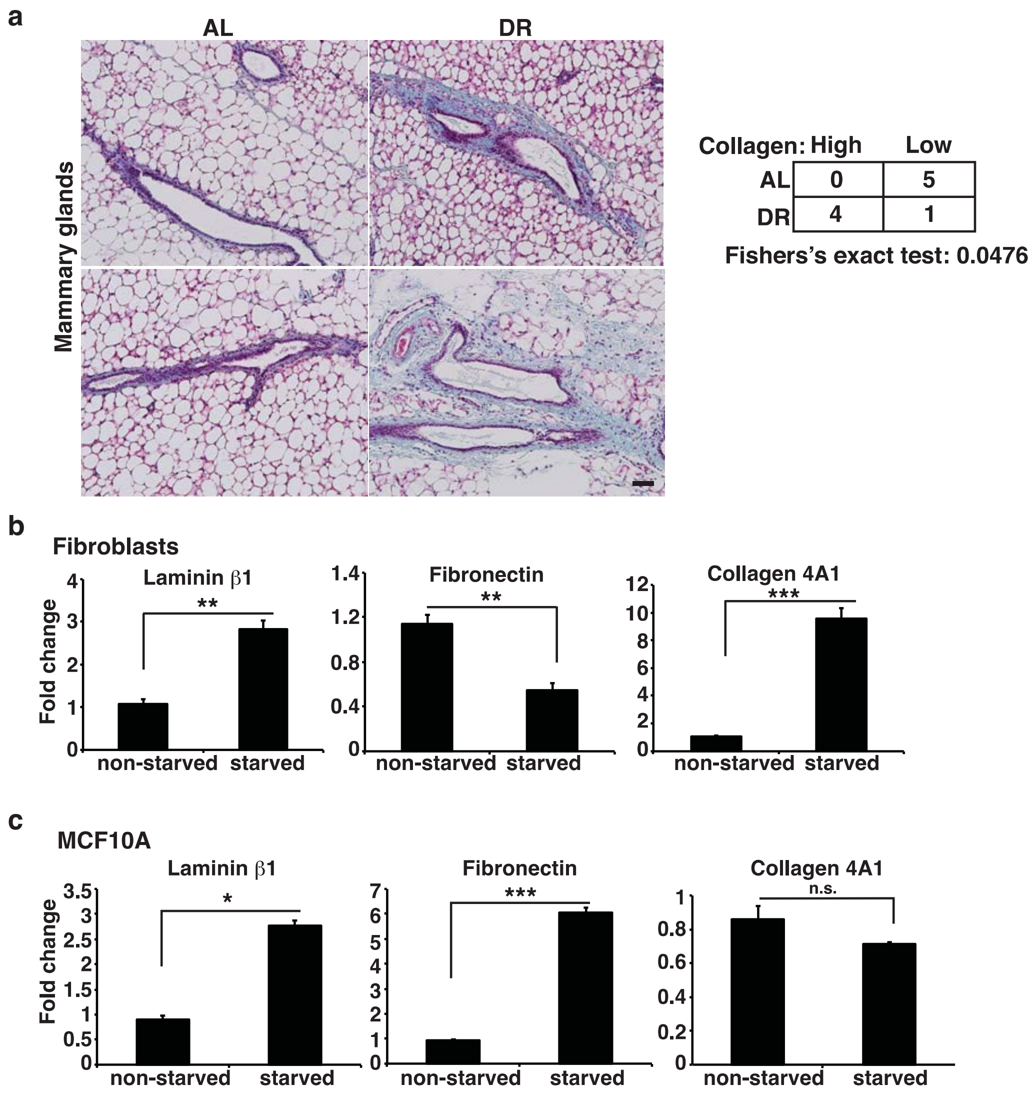

Dietary restricted mouse mammary glands have increased matrix secretion and starved human cells display increased extracellular matrix gene expression. (**a**) Trichrome staining of mammary glands from *ad-libitum*-fed (AL) or dietary restricted (DR) mice, with the amount of collagen (blue) scored blindly and analysed by Fisher's exact test; scale bar: 40μm. (**b,c**) QPCR analysis of extracellular matrix gene expression in primary human fibroblasts (**b**) and MCF10A cells (**c**) that were either kept in non-starvation or starvation medium for 5 days; each value represents the mean ± s.e.m.; n=3; Student's t-test, *p<0.05, **p<0.01, ***p<0.001; n.s., non-significant.

Supplementary Figure 6

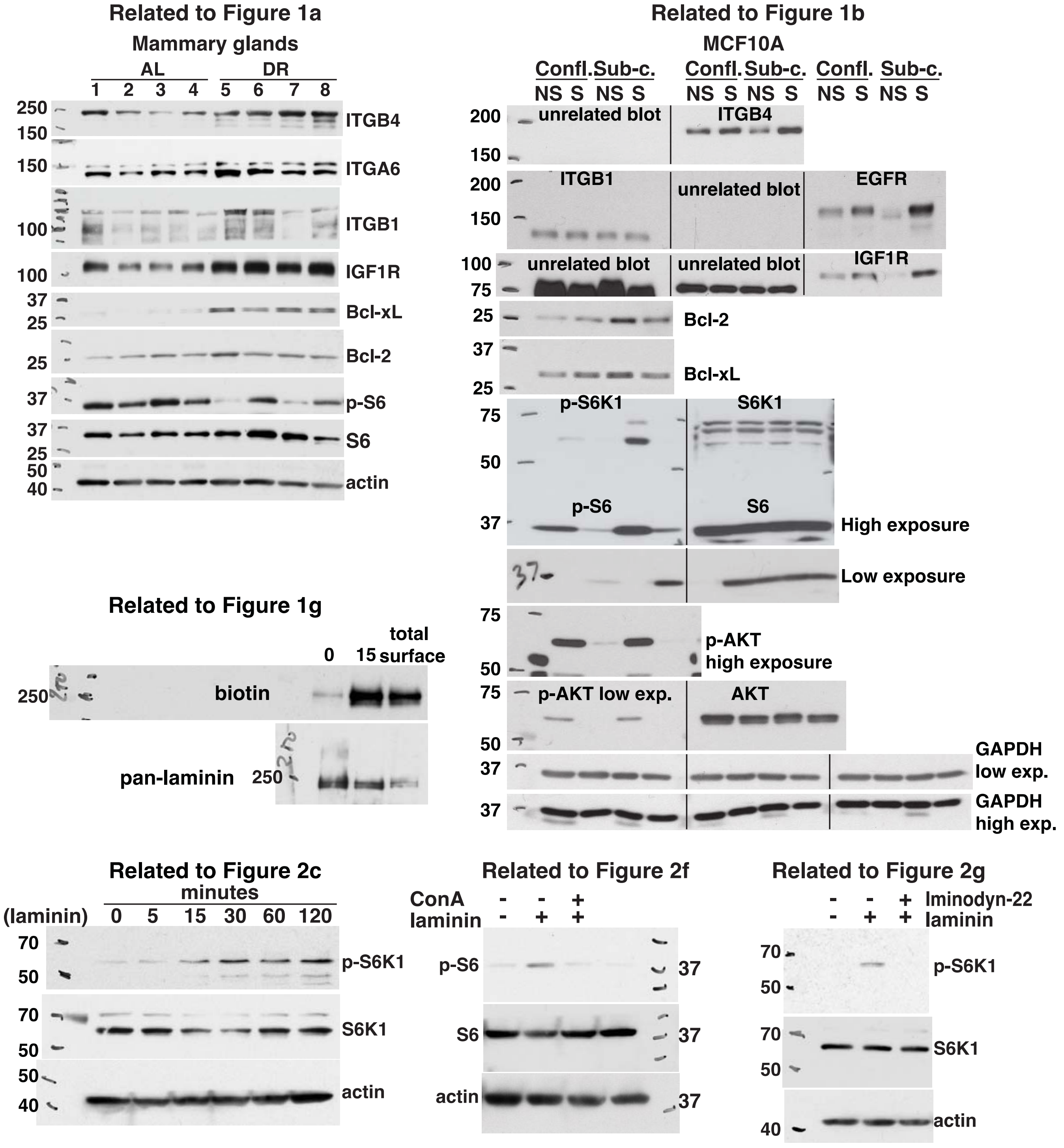

Scanned films of uncropped Western blots related to the indicated panels from Main Figures 1 and 2. Blots that were developped together on the same film are separated by a black line.

Supplementary Figure 7

Related to Figure 3a

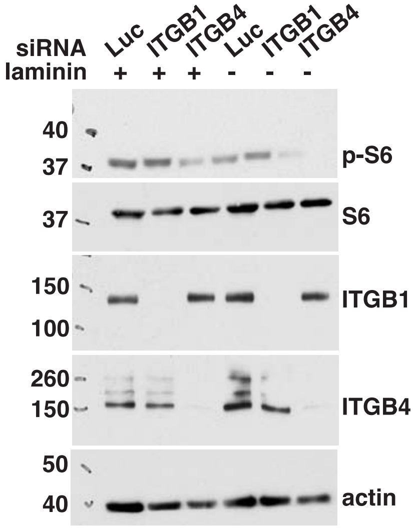

Related to Figure 3e

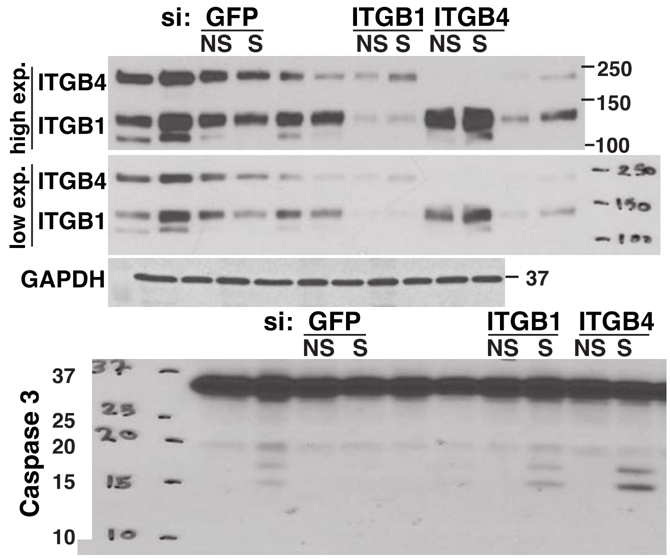

Related to Figure 5a

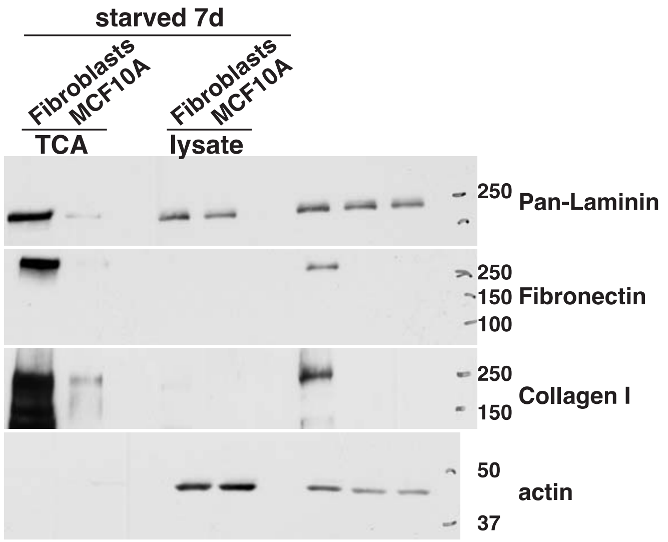

Related to Figure 5d

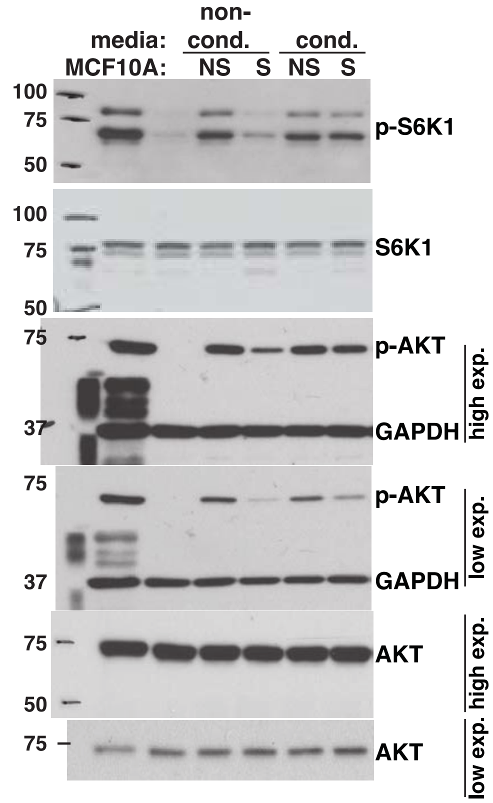

Scanned films of uncropped Western blots related to the indicated panels from Main Figures 3 and 5 (part I).

Supplementary Figure 8

Related to Figure 5e

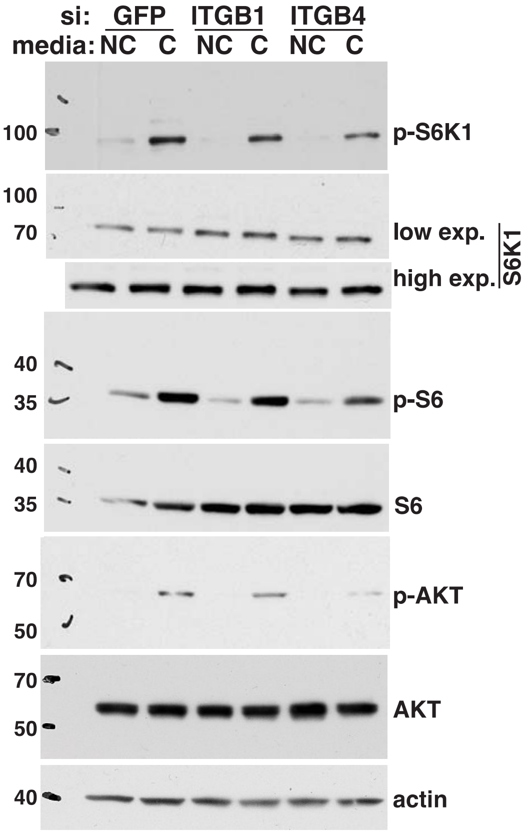

Related to Figure 5g

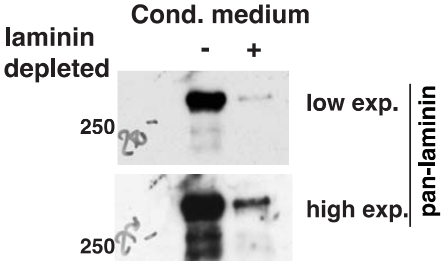

Related to Figure 5h

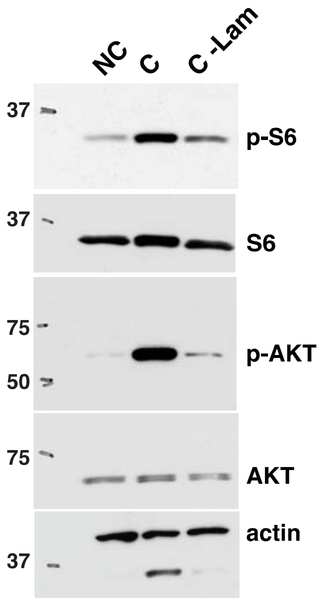

Related to Figure 5j

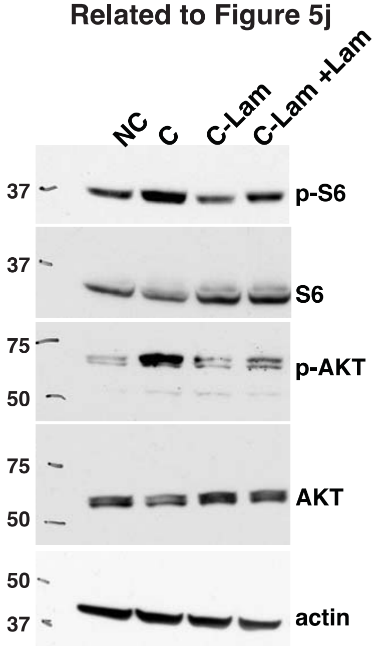

Scanned films of uncropped Western blots related to the indicated panels from Main Figure 5 (part II).

Supplementary Table 1

List of components in experimental culture media used to treat MCF10A cells

Media Components	non-starvation	starvation	AL	DR
**Horse serum**	5%	0%	0 %	0 %
**Fatty acid-free dialyzed albumin**	0%	0%	0.9 %	0.9 %
**Insulin**	10 μg/ml	0 μg/ml	1 μg/ml	1 ng/ml
**EGF**	20 ng/ml	0 ng/ml	20 ng/ml	0 ng/ml
**Glucose**	3151 mg/l	3151 mg/l	3151 mg/l	2363 mg/l
**Hydrocortisone**	0.5 μg/ml	0.5 μg/ml	0.5 μg/ml	0.5 μg/ml
**Cholera toxin**	0.1 μg/ml	0.1 μg/ml	0.1 μg/ml	0.1 μg/ml
**Penicillin-Streptomycin**	50 U/ml-50 μg/ml	50 U/ml-50 μg/ml	50 U/ml-50 μg/ml	50 U/ml-50 μg/ml
**Alanine**	4.45 mg/l	4.45 mg/l	4.45 mg/l	6.36 mg/l
**Isoleucine**	54.47 mg/l	54.47 mg/l	54.47 mg/l	32.68 mg/l
**Leucine**	59.05 mg/l	59.05 mg/l	59.05 mg/l	38.35 mg/l
**Lysine**	91.25 mg/l	91.25 mg/l	91.25 mg/l	60.22 mg/l
**Methionine**	17.24 mg/l	17.24 mg/l	17.24 mg/l	34.48 mg/l
**Serine**	26.25 mg/l	26.25 mg/l	26.25 mg/l	36.48 mg/l
**Tyrosine**	55.79 mg/l	55.79 mg/l	55.79 mg/l	34.03 mg/l
**Valine**	52.85 mg/l	52.85 mg/l	52.85 mg/l	34.35 mg/l
**All other media components are similar to those in DMEM:F12 (Invitrogen #11330)**				
